# Supplementary material for: Measuring algorithmic bias to analyze the reliability of AI tools that predict depression risk using smartphone sensed-behavioral data
Source: Npj Ment Health Res. 2024 Apr 22;3:17. doi: 10.1038/s44184-024-00057-y (PMC11035598; doi:10.1038/s44184-024-00057-y)
Supplement: Supplementary file 1 — Reporting summary [file 44184_2024_57_MOESM1_ESM.pdf]

nature portfolio

Corresponding author(s): Daniel A. Adler

Last updated by author(s): February 18, 2024

Reporting Summary

Nature Portfolio wishes to improve the reproducibility of the work that we publish. This form provides structure for consistency and transparency in reporting. For further information on Nature Portfolio policies, see our Editorial Policies and the Editorial Policy Checklist.

Please do not complete any field with "not applicable" or n/a. Refer to the help text for what text to use if an item is not relevant to your study.
For final submission: please carefully check your responses for accuracy; you will not be able to make changes later.

Statistics

For all statistical analyses, confirm that the following items are present in the figure legend, table legend, main text, or Methods section.

n/a	Confirmed	
☐	☒	The exact sample size (*n*) for each experimental group/condition, given as a discrete number and unit of measurement
☐	☒	A statement on whether measurements were taken from distinct samples or whether the same sample was measured repeatedly
☒	☐	The statistical test(s) used AND whether they are one- or two-sided<br>*Only common tests should be described solely by name; describe more complex techniques in the Methods section.*
☐	☒	A description of all covariates tested
☐	☒	A description of any assumptions or corrections, such as tests of normality and adjustment for multiple comparisons
☐	☒	A full description of the statistical parameters including central tendency (e.g. means) or other basic estimates (e.g. regression coefficient) AND variation (e.g. standard deviation) or associated estimates of uncertainty (e.g. confidence intervals)
☒	☐	For null hypothesis testing, the test statistic (e.g. *F*, *t*, *r*) with confidence intervals, effect sizes, degrees of freedom and *P* value noted<br>*Give P values as exact values whenever suitable.*
☒	☐	For Bayesian analysis, information on the choice of priors and Markov chain Monte Carlo settings
☒	☐	For hierarchical and complex designs, identification of the appropriate level for tests and full reporting of outcomes
☐	☒	Estimates of effect sizes (e.g. Cohen's *d*, Pearson's *r*), indicating how they were calculated

Our web collection on statistics for biologists contains articles on many of the points above.

Software and code

Policy information about availability of computer code

Data collection	This was a secondary analysis of collected data.
Data analysis	Python version 3.8.10 with the following libraries: Pandas (v1.2.5), Numpy (v1.20.2), Seaborn (v0.12.2), Pingouin (v0.5.3), Sklearn (v1.0.2), Matplotlib (v3.6.2), Statsmodels (v0.13.5), Scipy (v1.10.1),

For manuscripts utilizing custom algorithms or software that are central to the research but not yet described in published literature, software must be made available to editors and reviewers. We strongly encourage code deposition in a community repository (e.g. GitHub). See the Nature Portfolio guidelines for submitting code & software for further information.

Data

Policy information about availability of data

All manuscripts must include a data availability statement. This statement should provide the following information, where applicable:

- Accession codes, unique identifiers, or web links for publicly available datasets
- A description of any restrictions on data availability
- For clinical datasets or third party data, please ensure that the statement adheres to our policy

Sensed-behavioral data cannot be made publicly available due to potentially identifying information (e.g. GPS location) that may compromise participant privacy. De-identified self-reported data (the PHQ-8) will be made available through the NIMH Data Archive.

Research involving human participants, their data, or biological material

Policy information about studies with human participants or human data. See also policy information about sex, gender (identity/presentation), and sexual orientation and race, ethnicity and racism.

Reporting on sex and gender	We analyzed the variable "sex at birth", which was obtained from participants who self-reported this information after being prompted with the question: "Assigned sex at birth: What sex were you assigned at birth, on your original birth certificate?". We were interested in this variable, as our study focused on analyzing differences in sensed-behaviors, and how these differences affect AI tools, across individuals from different demographic and socioeconomic variables, including sex at birth.<br><br>This is a secondary analysis of data, so the choice of collecting sex at birth in this method was out of scope for this work.
Reporting on race, ethnicity, or other socially relevant groupings	In addition to sex at birth, we analyzed "Age", "Family Income", "Health Insurance Status", "Race", and "Employment Status". We were interested in these variables, as our study focused on analyzing differences in sensed-behaviors, and how these differences affect AI tools, across individuals from different demographic and socioeconomic variables. This information was provided by participant self-reports, in response to the following questions:<br><br>Age: What is your age?<br>Family Income: What is your best estimate of your combined family income from all sources, before taxes, in the last calendar year?<br>Health Insurance Status: Are you covered by health insurance or some other kind of health care plan?<br>Race: What is your race?<br>Employment Status: What is your current employment status? (If more than one category applies, and you are working in some capacity, please select "Employed/Self-Employed")<br><br>This is a secondary analysis of data, so the choice of collecting these variables in this method was out of scope for this work.
Population characteristics	We specifically describe the data analyzed in this work:<br><br>Total number of participants: 650<br><br>Age - 18 to 25: 60<br>Age - 25 to 34: 181<br>Age - 35 to 44: 168<br>Age - 45 to 54: 135<br>Age - 55 to 64: 81<br>Age - 65 to 74: 22<br>Age - 75 to 84: 3<br><br>Sex at Birth - Female: 482<br>Sex at Birth - Male: 168<br><br>Family Income - <20,000: 98<br>Family Income - 20,000 to 39,999: 144<br>Family Income - 40,000 to 59,999: 124<br>Family Income - 60,000 to 99,999: 161<br>Family Income - 100,000+: 110<br>Family Income - Don't know: 10<br>Family Income - Prefer not to answer: 3<br><br>Health Insurance Status - Insured: 603<br>Health Insurance Status - Uninsured: 43<br>Health Insurance Status - Don't know: 3<br>Health Insurance Status - Prefer not to answer: 1<br><br>Race - White: 534<br>Race - Black/African American: 61<br>Race - Asian/Asian American: 22<br>Race - More than one race: 24<br>Race - Other: 6<br>Race - Prefer not to answer: 3<br><br>Employment Status - Employed: 401<br>Employment Status - Unemployed: 90<br>Employment Status - Disability: 72<br>Employment Status - Retired: 34<br>Employment Status - Other: 52<br>Employment Status - Prefer not to answer: 1
Recruitment	Participants were recruited from across the United States using digital registries and online advertisements, intentionally oversampling for individuals experiencing depression. Eligible participants lived in the United States, could read/write English, and owned an Android smartphone and data plan. In addition, eligible participants with at least moderate depression symptom severity based upon the Patient Health Questionnaire-8 (PHQ-8) ≥10 were oversampled to create a sample with

elevated symptoms. Individuals were excluded from the study if they self-reported a diagnosis of bipolar disorder, any psychotic disorder, shared a smartphone with another individual, or were unwilling to share data. Eligible participants were asked to provide electronic informed consent after receiving a complete description of the study. Eligible participants had the option to not provide consent, and could withdraw from the study at any point.

This is a secondary analysis of data, so the recruitment and eligibility criteria are out of scope for this work.

Ethics oversight	Study procedures were approved by the Northwestern University Institutional Review Board (IRB study #STU00205316). Given this is a secondary analysis of data, datasets were provided after appropriate agreements were put in place across the coauthors' institutions to rely on the Northwestern IRB.

Note that full information on the approval of the study protocol must also be provided in the manuscript.

Field-specific reporting

Please select the one below that is the best fit for your research. If you are not sure, read the appropriate sections before making your selection.

☐ Life sciences ☒ Behavioural & social sciences ☐ Ecological, evolutionary & environmental sciences

Behavioural & social sciences study design

All studies must disclose on these points even when the disclosure is negative.

Study description	The data was collected during a prospective study to analyze the relationships between sensed-behavioral data and symptoms of major depressive disorder.
Research sample	Participants were recruited from across the United States using digital registries and online advertisements, intentionally oversampling for individuals experiencing depression. Eligible participants lived in the United States, could read/write English, and owned an Android smartphone and data plan. Individuals were excluded from the study if they self-reported a diagnosis of bipolar disorder, any psychotic disorder, shared a smartphone with another individual, or were unwilling to share data. Datasets were shared across researchers interested in studying the reliability of sensed-behaviors to predict depression symptoms after appropriate agreements were put in place to rely on the Northwestern IRB.
Sampling strategy	Eligible participants with at least moderate depression symptom severity based upon the Patient Health Questionnaire-8 (PHQ-8) ≥10 were oversampled to create a sample with elevated symptoms (50% PHQ-8 ≥10).
Data collection	An Android application was developed for the purposes of this study to passively collect sensed-behavioral data and administer self-reports. In addition, self-reports were also collected during each weekly reporting period (study weeks 1, 4, 7, etc.) using REDCap. Data collection took place for 16 weeks, and self-reports were administered via both REDCap and the Android application every three weeks beginning on week 1 of the study.
Timing	Data was collected over the following (mm-dd-yy):<br><br>Wave 1 Initial Deployment: 05-06-19 to 08-25-19<br>Wave 1 Main Deployment: 07-30-19 to 11-18-19<br>Wave 2 Group 1: 02-11-20 to 06-01-20<br>Wave 2 Group 2: 04-14-20 to 08-03-20<br>Wave 3 Group 1: 01-19-21 to 05-10-21<br>Wave 3 Group 2: 02-09-21 to 05-31-21<br>Wave 3 Group 3: 04-13-21 to 08-02-21
Data exclusions	Data was excluded from individuals who did not self-report at least one PHQ-8 depression symptom survey during each of the 6 weekly reporting periods such that our results would not be biased by the number of self-reports obtained from individual participants.
Non-participation	Given this was a secondary analysis we did not observe, use, and analyze data from participants who dropped out of the study.
Randomization	Not applicable, no randomization.

Reporting for specific materials, systems and methods

We require information from authors about some types of materials, experimental systems and methods used in many studies. Here, indicate whether each material, system or method listed is relevant to your study. If you are not sure if a list item applies to your research, read the appropriate section before selecting a response.

Materials & experimental systems

n/a	Involved in the study
☒	☐ Antibodies
☒	☐ Eukaryotic cell lines
☒	☐ Palaeontology and archaeology
☒	☐ Animals and other organisms
☒	☐ Clinical data
☒	☐ Dual use research of concern
☒	☐ Plants

Methods

n/a	Involved in the study
☒	☐ ChIP-seq
☒	☐ Flow cytometry
☒	☐ MRI-based neuroimaging

This checklist template is licensed under a Creative Commons Attribution 4.0 International License, which permits use, sharing, adaptation, distribution and reproduction in any medium or format, as long as you give appropriate credit to the original author(s) and the source, provide a link to the Creative Commons license, and indicate if changes were made. The images or other third party material in this article are included in the article's Creative Commons license, unless indicated otherwise in a credit line to the material. If material is not included in the article's Creative Commons license and your intended use is not permitted by statutory regulation or exceeds the permitted use, you will need to obtain permission directly from the copyright holder. To view a copy of this license, visit http://creativecommons.org/licenses/by/4.0/
