# Supplementary material for: Measuring algorithmic bias to analyze the reliability of AI tools that predict depression risk using smartphone sensed-behavioral data
Source: Npj Ment Health Res. 2024 Apr 22;3:17. doi: 10.1038/s44184-024-00057-y (PMC11035598; doi:10.1038/s44184-024-00057-y)
Supplement: Supplementary file 2 — Supplementary Information [file 44184_2024_57_MOESM2_ESM.docx]

# Supplementary Materials

**Supplementary Figure 1. Analyzing bias across models.** Bias was assessed by measuring the area under the receiver operating curve of positive and negative samples within subgroups (Subgroup AUC, left column), subgroup positive samples compared to negative samples from the background (BNSP AUC, middle column, the “background” is all individuals not in the subgroup), and subgroup negative samples compared to background positive samples (BPSN AUC, right column)^1,2^. Bar heights indicate the median across trials, and error bars show 95% confidence intervals (2.5 and 97.5 percentiles).


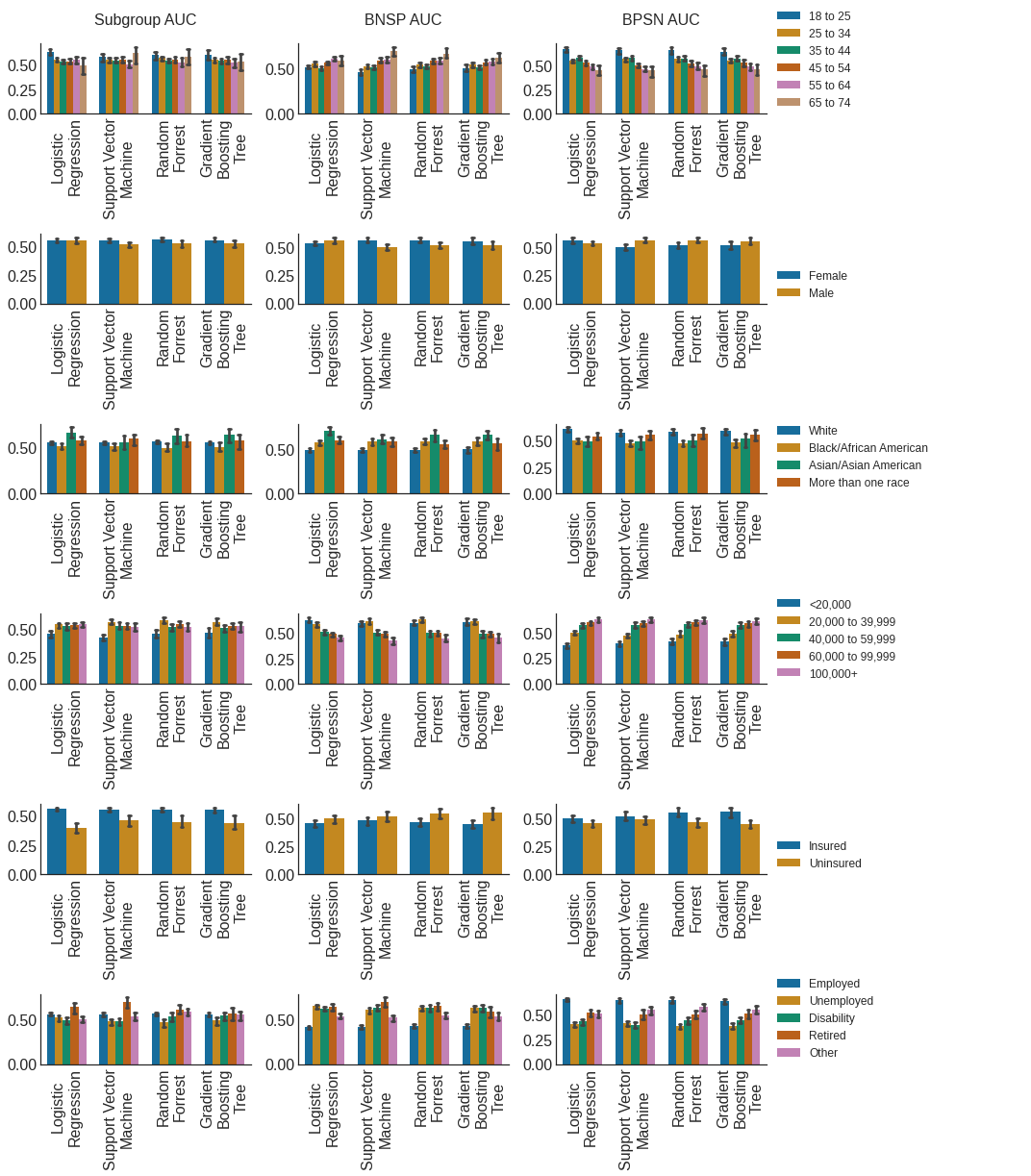


**Supplementary Figure 2. Prevalence and BNSP/BPSN AUC.** The left column shows the prevalence (% samples experiencing clinically-significant depression) across subgroups. The right two columns show the background-negative-subgroup-positive (BNSP), and background-positive-subgroup-negative AUCs. Bar heights/point values are the median values across trials and error bars represent 95% confidence intervals (2.5 and 97.5 percentiles). Dotted lines and shaded areas show the distribution (median and 95% confidence intervals) of either the median (if >2 subgroups) or highest performing subgroup across trials.


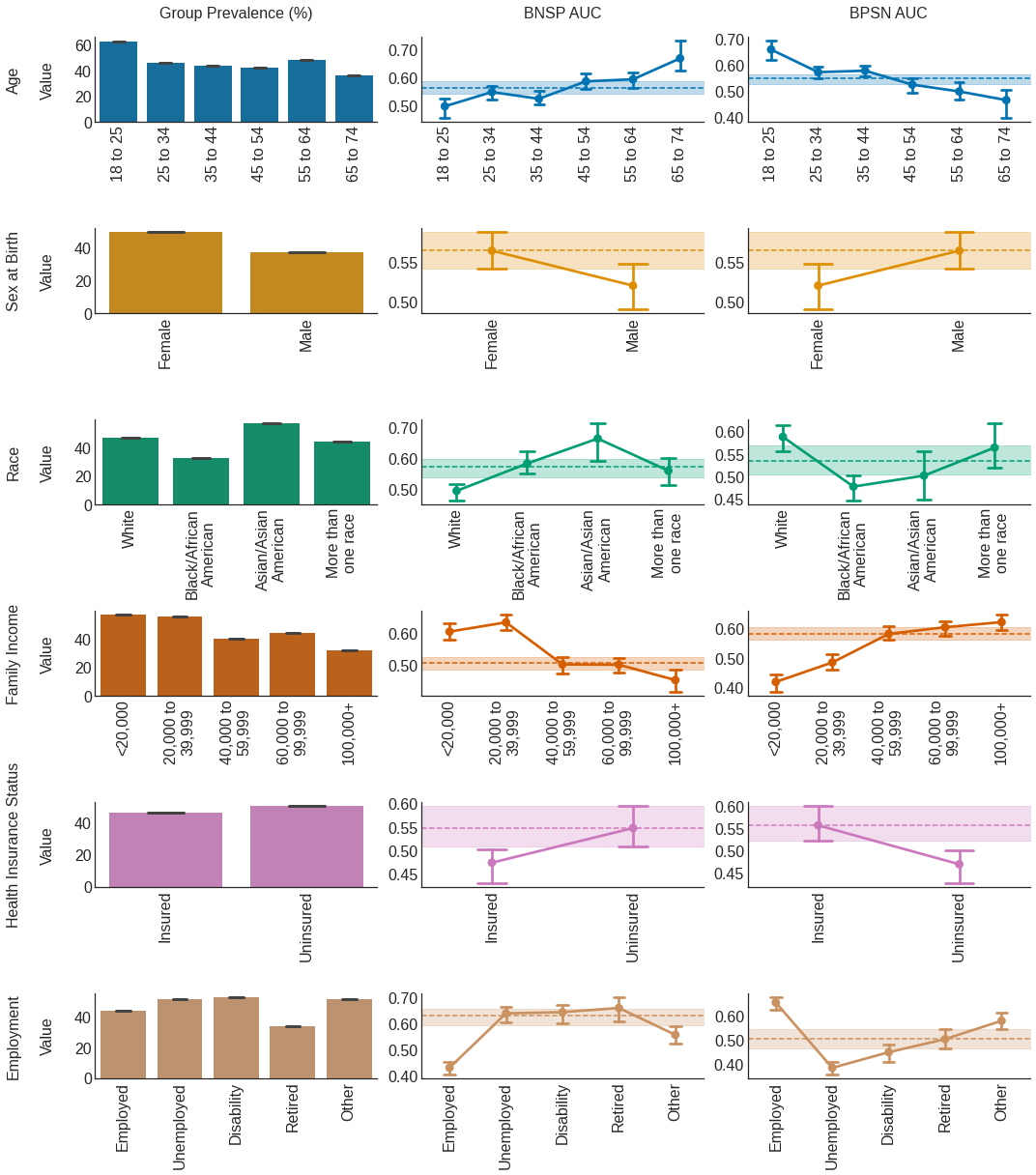


**Supplementary Figure 3. Number of samples and the Subgroup AUC.** The left column shows the log number of samples across subgroups. The right column shows the subgroup area under the receiver operating curve (Subgroup AUC). Bar heights/point values are the median values across trials and error bars represent 95% confidence intervals (2.5 and 97.5 percentiles).


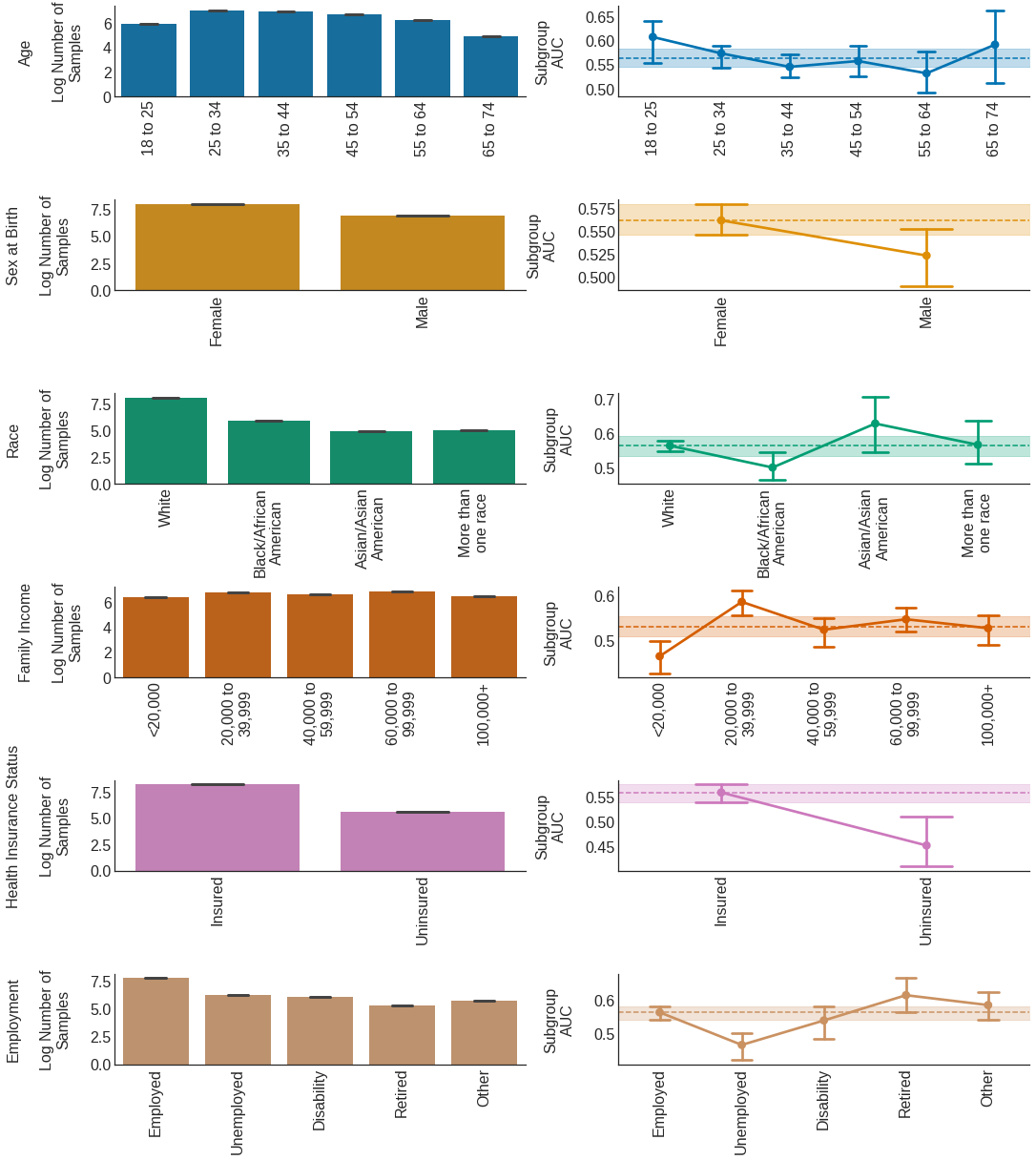


**Supplementary Tables 1. Relationships between sensed-behavioral features and depression across specific subgroups.** Values are listed only for features where subgroups had significantly different relationships (Bonferroni corrected α’ =α / number of comparisons, α = 0.05) between sensed-behaviors and depression. α’ is listed above each table. β was calculated by adding the effect associated with the majority subgroup plus the difference in the effect from being a member of a non-majority subgroup (an interaction term between subgroup membership and the feature value), or for majority subgroups, is simply the majority subgroup effect. The median feature values per subgroup, with 95% confidence intervals (CIs: 2.5% and 97.5% percentiles) are also included. All values are standardized units.

α’=0.008

| **Age** | **Sensed-Behavior** | **β (95% CI)** | **Median Feature Value (95% CI)** |
| --- | --- | --- | --- |
| 25 to 34 | Avg Phone Unlocks 12-6PM | –0.21 (–0.33 to –0.09) | 0.38 (–2.69 to 1.63) |
| 65 to 74 | Avg Phone Unlocks 12-6PM | 0.68 (0.14 to 1.22) | –0.79 (–2.55 to 0.37) |
| 18 to 25 | Avg Phone Unlocks 6-12PM | –0.77 (–1.07 to –0.47) | 0.32 (–2.27 to 1.60) |
| 25 to 34 | Avg Phone Unlocks 6-12PM | –0.24 (–0.36 to –0.12) | 0.18 (–2.27 to 1.86) |
| 65 to 74 | Avg Phone Unlocks 6-12PM | 0.60 (0.07 to 1.12) | –0.62 (–1.96 to 0.76) |
| 25 to 34 | Avg Phone Unlocks Entire Day | –0.17 (–0.29 to –0.06) | 0.37 (–2.71 to 1.56) |
| 65 to 74 | Avg Phone Unlocks Entire Day | 0.74 (0.19 to 1.29) | –0.68 (–2.39 to 0.38) |
| 45 to 54 | Avg Phone Unlock Duration 6-12AM | –0.40 (–0.59 to –0.22) | 0.03 (–2.12 to 2.20) |
| 65 to 74 | Avg Phone Unlock Duration 6-12AM | 0.59 (0.17 to 1.01) | 0.04 (–2.68 to 1.57) |
| 45 to 54 | Avg Phone Unlock Duration Entire Day | –0.30 (–0.48 to –0.12) | 0.10 (–2.23 to 1.92) |
| 65 to 74 | Avg Phone Unlock Duration Entire Day | 0.65 (0.23 to 1.07) | 0.12 (–2.66 to 1.24) |
| 25 to 34 | Deviation in Phone Unlocks 6-12AM | –0.22 (–0.35 to -0.10) | 0.33 (–2.00 to 2.01) |
| 35 to 44 | Deviation in Phone Unlocks 6-12AM | 0.18 (0.01 to 0.36) | 0.20 (–2.21 to 1.99) |
| 18 to 25 | Deviation in Phone Unlocks Entire Day | –0.64 (–0.95 to –0.33) | 0.41 (–2.29 to 1.68) |
| 25 to 34 | Deviation in Phone Unlocks Entire Day | –0.20 (–0.32 to –0.08) | 0.28 (–2.35 to 1.90) |
| 45 to 54 | Deviation Phone Unlock Duration 6-12AM | –0.37 (–0.56 to –0.18) | –0.06 (–1.70 to 2.16) |
| 65 to 74 | Deviation Phone Unlock Duration 6-12AM | 0.67 (0.23 to 1.11) | 0.09 (–2.34 to 1.42) |
| 45 to 54 | Deviation Phone Unlock Duration Entire Day | –0.31 (–0.49 to –0.12) | –0.07 (–1.66 to 2.32) |
| 65 to 74 | Deviation Phone Unlock Duration Entire Day | 0.76 (0.26 to 1.26) | –0.04 (–1.93 to 1.28) |

α’=0.025

| **Sex at Birth** | **Sensed-Behavior** | **β (95% CI)** | **Median Feature Value (95% CI)** |
| --- | --- | --- | --- |
| Female | Avg Phone Unlocks Entire Day | –0.10 (–0.18 to –0.03) | 0.18 (–2.60 to 1.51) |
| Male | Avg Phone Unlocks Entire Day | 0.16 (0.01 to 0.30) | 0.15 (–2.84 to 1.71) |
| Female | Days Phone Unlocked from 12-6AM | 0.12 (0.05 to 0.19) | 0.06 (–1.92 to 1.26) |
| Male | Days Phone Unlocked from 12-6AM | 0.30 (0.15 to 0.46) | 0.27 (–1.92 to 1.26) |
| Female | Deviation in Phone Unlocks 12-6PM | –0.10 (–0.17 to –0.02) | 0.07 (–2.24 to 1.77) |
| Male | Deviation in Phone Unlocks 12-6PM | 0.18 (0.04 to 0.32) | 0.03 (–2.84 to 1.73) |

α’=0.0125

| **Race** | **Sensed-Behavior** | **β (95% CI)** | **Median Feature Value (95% CI)** |
| --- | --- | --- | --- |
| Asian/Asian American | Avg Phone Unlocks 12-6PM | –1.06 (–1.62 to –0.50) | 0.23 (–1.81 to 1.14) |
| More than one race | Avg Phone Unlocks 12-6PM | –0.53 (–0.90 to –0.15) | 0.18 (–2.10 to 1.69) |
| White | Avg Phone Unlocks 6-12PM | –0.11 (–0.18 to –0.04) | 0.06 (–2.16 to 1.80) |
| Asian/Asian American | Avg Phone Unlocks 6-12PM | –1.56 (–2.23 to –0.89) | –0.07 (–2.23 to 1.09) |
| Asian/Asian American | Avg Phone Unlocks Entire Day | –0.91 (–1.51 to –0.31) | 0.20 (–1.94 to 1.05) |
| More than one race | Avg Phone Unlocks Entire Day | –0.47 (–0.84 to –0.10) | 0.23 (–2.70 to 1.73) |
| White | Deviation Phone Unlock Duration 6-12PM | –0.09 (–0.16 to –0.02) | –0.13 (–1.61 to 2.78) |
| More than one race | Deviation Phone Unlock Duration 6-12PM | 0.52 (0.05 to 1.00) | 0.00 (–1.05 to 2.21) |

α’=0.01

| **Family Income** | **Sensed-Behavior** | **β (95% CI)** | **Median Feature Value (95% CI)** |
| --- | --- | --- | --- |
| <20,000 | % Time at Home | –0.30 (–0.52 to –0.08) | 0.44 (–3.97 to 0.83) |
| 60,000 to 99,999 | % Time at Home | 0.42 (0.26 to 0.58) | 0.18 (–2.80 to 0.82) |
| 40,000 to 59,999 | Avg Phone Unlocks 12-6AM | –0.30 (–0.51 to –0.08) | –0.17 (–1.12 to 2.02) |
| 100,000+ | Avg Phone Unlocks 12-6AM | 0.41 (0.17 to 0.65) | –0.38 (–1.71 to 1.90) |
| <20,000 | Circadian Movement | 0.30 (0.09 to 0.51) | –0.19 (–2.65 to 1.67) |
| 60,000 to 99,999 | Circadian Movement | –0.21 (–0.35 to –0.07) | 0.29 (–1.89 to 1.82) |
| <20,000 | Location Entropy | 0.35 (0.14 to 0.57) | –0.37 (–1.21 to 2.87) |
| 60,000 to 99,999 | Location Entropy | –0.34 (–0.48 to –0.20) | 0.04 (–1.17 to 2.28) |
| <20,000 | Location Normalized Entropy | 0.36 (0.14 to 0.58) | –0.19 (–1.28 to 2.95) |
| 60,000 to 99,999 | Location Normalized Entropy | –0.33 (–0.48 to –0.18) | 0.04 (–1.23 to 2.08) |
| <20,000 | Location Variance | 0.36 (0.14 to 0.58) | –0.32 (–2.48 to 2.13) |
| 60,000 to 99,999 | Location Variance | –0.21 (–0.35 to –0.06) | 0.12 (–1.56 to 2.37) |

α’=0.025

| **Health Insurance Status** | **Sensed-Behavior** | **β (95% CI)** | **Median Feature Value (95% CI)** |
| --- | --- | --- | --- |
| Insured | % Location Data in Transition | –0.15 (–0.22 to –0.08) | –0.26 (–1.11 to 2.44) |
| Uninsured | % Location Data in Transition | 0.32 (0.11 to 0.52) | –0.16 (–1.10 to 4.44) |
| Insured | % Time at Home | 0.18 (0.11 to 0.25) | 0.36 (–2.86 to 0.83) |
| Uninsured | % Time at Home | –0.26 (–0.46 to –0.06) | 0.13 (–3.97 to 0.82) |
| Insured | Avg Phone Unlocks 12-6PM | –0.08 (–0.14 to –0.01) | 0.11 (–2.36 to 1.66) |
| Uninsured | Avg Phone Unlocks 12-6PM | 0.31 (0.04 to 0.57) | 0.19 (–2.26 to 1.99) |
| Insured | Avg Phone Unlocks 6-12PM | –0.18 (–0.24 to –0.11) | 0.09 (–2.18 to 1.82) |
| Uninsured | Avg Phone Unlocks 6-12PM | 0.31 (0.06 to 0.56) | –0.13 (–2.15 to 2.18) |
| Insured | Deviation in Phone Unlocks 6-12PM | –0.10 (–0.17 to –0.03) | 0.06 (–2.29 to 1.82) |
| Uninsured | Deviation in Phone Unlocks 6-12PM | 0.29 (0.04 to 0.55) | 0.04 (–2.09 to 1.85) |

α’=0.01

| **Employment** | **Sensed-Behavior** | **β (95% CI)** | **Median Feature Value (95% CI)** |
| --- | --- | --- | --- |
| Disability | Avg Phone Unlocks 12-6AM | –0.22 (–0.44 to –0.01) | –0.14 (–1.71 to 2.27) |
| Retired | Avg Phone Unlocks 12-6AM | 1.05 (0.55 to 1.56) | –0.52 (–1.15 to 1.05) |
| Employed | Avg Phone Unlocks 12-6AM | 0.15 (0.06 to 0.23) | –0.22 (–1.12 to 2.15) |
| Other | Avg Phone Unlock Duration 12-6AM | –0.39 (–0.65 to –0.13) | –0.04 (–2.25 to 1.56) |
| Disability | Avg Phone Unlock Duration 12-6AM | –0.57 (–0.82 to –0.33) | 0.25 (–2.21 to 1.77) |
| Retired | Avg Phone Unlock Duration 12-6AM | 0.59 (0.24 to 0.95) | –0.01 (–2.21 to 1.21) |
| Disability | Avg Phone Unlock Duration 6-12AM | –0.33 (–0.55 to –0.12) | 0.19 (–2.32 to 1.74) |
| Other | Avg Phone Unlock Duration 6-12AM | –0.54 (–0.86 to –0.21) | 0.07 (–1.70 to 1.66) |
| Disability | Avg Phone Unlock Duration 6-12PM | –0.51 (–0.75 to –0.27) | 0.13 (–2.48 to 1.79) |
| Retired | Avg Phone Unlock Duration 6-12PM | 0.38 (0.06 to 0.70) | 0.11 (–2.16 to 1.88) |
| Employed | Avg Phone Unlock Duration 6-12PM | –0.11 (–0.19 to –0.03) | –0.02 (–1.81 to 2.49) |
| Disability | Avg Phone Unlock Duration Entire Day | –0.45 (–0.68 to –0.22) | 0.21 (–2.53 to 1.58) |
| Other | Avg Phone Unlock Duration Entire Day | –0.60 (–0.94 to –0.25) | 0.16 (–1.47 to 1.54) |
| Retired | Avg Phone Unlock Duration Entire Day | 0.48 (0.16 to 0.79) | –0.05 (–2.67 to 1.45) |
| Employed | Circadian Movement | –0.16 (–0.24 to –0.07) | 0.20 (–2.06 to 2.09) |
| Disability | Circadian Movement | 0.44 (0.21 to 0.66) | –0.20 (–2.34 to 1.53) |
| Retired | Days Phone Unlocked from 12-6AM | 1.30 (0.87 to 1.73) | –0.15 (–1.92 to 1.26) |
| Employed | Days Phone Unlocked from 12-6AM | 0.20 (0.12 to 0.28) | 0.06 (–1.92 to 1.26) |
| Employed | Deviation in Phone Unlocks 12-6AM | 0.16 (0.07 to 0.24) | –0.15 (–1.45 to 2.07) |
| Disability | Deviation in Phone Unlocks 12-6AM | –0.28 (–0.50 to –0.07) | –0.09 (–1.55 to 2.10) |
| Retired | Deviation in Phone Unlocks 12-6AM | 0.94 (0.48 to 1.41) | –0.51 (–1.45 to 0.90) |
| Retired | Deviation Phone Unlock Duration 12-6AM | 0.54 (0.19 to 0.89) | –0.03 (–2.98 to 1.19) |
| Other | Deviation Phone Unlock Duration 12-6AM | –0.40 (–0.64 to –0.15) | –0.03 (–2.93 to 1.51) |
| Disability | Deviation Phone Unlock Duration 12-6AM | –0.52 (–0.77 to –0.27) | 0.27 (–2.37 to 1.57) |
| Unemployed | Deviation Phone Unlock Duration 12-6PM | 0.23 (0.04 to 0.42) | –0.19 (–1.65 to 2.76) |
| Disability | Deviation Phone Unlock Duration 12-6PM | –0.34 (–0.57 to –0.11) | –0.03 (–1.47 to 2.38) |
| Disability | Location Variance | 0.47 (0.24 to 0.69) | –0.31 (–2.15 to 1.96) |
| Employed | Location Variance | –0.16 (–0.25 to –0.08) | 0.05 (–1.86 to 2.46) |
| Disability | Number of Locations | 0.23 (0.02 to 0.43) | –0.09 (–2.09 to 1.60) |
| Employed | Number of Locations | –0.17 (–0.26 to –0.09) | 0.13 (–2.09 to 1.71) |

**Supplementary Workbook.** The supplementary workbook (“Supplementary Workbook on Sensed-Behaviors.xlsx”) contains, for each attribute, a sheet that shows the median and interquartile ranges (25th to 75th percentiles) of each feature value across the subgroups.

**References**

1. Borkan, D., Dixon, L., Sorensen, J., Thain, N. &amp; Vasserman, L. Nuanced Metrics for Measuring Unintended Bias with Real Data for Text Classification. In *Companion Proceedings of The 2019 World Wide Web Conference* 491–500 (ACM, 2019).

2. Vogel, R., Bellet, A. & Clémençon, S. Learning Fair Scoring Functions: Bipartite Ranking under ROC-based Fairness Constraints. in *Proceedings of The 24th International Conference on Artificial Intelligence and Statistics* 784–792 (PMLR, 2021).
